# The influence of fitness celebrity livestream on women’s exercise behavior intention: a moderated mediation model

**DOI:** 10.3389/fpsyg.2025.1683907

**Published:** 2025-11-24

**Authors:** Jianfeng He, Zijun Yao, Yuwen Shangguan, Huan Li

**Affiliations:** 1 Wushu College, Chengdu Sports University, Chengdu, China; 2 Social Science Research on the Sport and Exercise, Loughborough University, Loughborough, United Kingdom; 3 Department of Exercise Physiology, Kunsan National University, Jeollabuk-do, Republic of Korea; 4 Department of Articular Orthopaedics, The First People’s Hospital of Changzhou, The Third Affiliated Hospital of Soochow University, Changzhou, China

**Keywords:** exercise behavioral intentions, social presence, body satisfaction, women, celebrity livestream

## Abstract

**Objective:**

Utilizing the S-O-R model as a guide, this research delved into how celebrity characteristics and media content characteristics impact women’s behavioral intentions. The study zeroed in on the role of body satisfaction as a moderator in the relationship between social presence and women’s exercise behavioral intentions. The aim is to reveal the intrinsic mechanism of the influence of fitness stars’ live streaming on women’s exercise behavior intention and to provide theoretical support to enhance women’s exercise intention, improve physical health, and help the implementation of the national health strategy. This study provides important theoretical support for the implementation of the national health strategy.

**Methods:**

A cross-sectional questionnaire survey was administered to a convenience sample of 382 female participants. Data analysis was performed using SPSS and AMOS; reliability analysis was first conducted to assess measurement consistency, followed by confirmatory factor analysis to validate the measurement model. Common method bias was then assessed using Harman’s single-factor test. Finally, both regression analysis and structural equation modeling were employed to test the hypothesized relationships in the theoretical framework.

**Results:**

Social presence mediated the relationship between celebrity characteristics: expertise (*β* = 0.207, *p* < 0.001), trustworthiness, (*β* = 0.195, *p* < 0.001) attractiveness (*β* = 0.251, *p* < 0.001), media content characteristics: perceived usefulness (*β* = 0.253, *p* < 0.001), perceived ease of uses (*β* = 0.224, *p* < 0.001) and women’s exercise behavior intentions. Body satisfaction moderated the relationship between social presence and women’s exercise behavior intentions.

**Conclusion:**

This study provides key theoretical and practical contributions by validating social presence as a mediator and body satisfaction as a moderator in the relationship between live-streaming stimuli and women’s behavioral intentions.

## 1 Introduction

The WHO ranks physical inactivity as the fourth major global mortality risk and a key 21st-century public health concern (Bull et al., 2020). In China, a populous country, nearly one-third of the population is physically inactive (Chen et al., 2023; Wang et al., 2021). As obesity and the prevalence of chronic diseases continue to rise, the exploration of innovative approaches to health promotion has become increasingly significant (Wang et al., 2023). Among various strategies, physical exercise is a cost-effective and accessible way to enhance health outcomes (Sallis, 2009), particularly for women, who have limited access to resources due to family care, and holds special value (Baker et al., 2002). However, the fast-paced nature of modern life, along with the time, skill, and environmental demands of traditional exercise methods, has made it challenging to increase women’s participation in exercise (Kimm et al., 2006). Thus, there is a growing need to enhance exercise intention in daily life while achieving health benefits comparable to or exceeding those of traditional forms of exercise.

Fitness livestreaming, which involves real-time demonstrations and interactive workouts conducted by fitness influencers on internet platforms such as Xiaohongshu, Douyin, Bilibili, and Kuaishou, has emerged as a novel exercise modality. This approach not only improves participants’ health but also overcomes the dual constraints of time and physical space associated with traditional exercise. Through professional and highly tailored content production, it has facilitated the equitable distribution of exercise resources. Its immersive and interactive experience has garnered widespread popularity, especially among Chinese women (Yu and Song, 2024).

In recent years, fitness livestreaming has demonstrated significant communicative potential and social influence. For instance, Chinese celebrity fitness influencer Liu Genghong went viral for his “Herbal Fitness Dance,” gaining over 70 million followers within a month and inspiring a trend among his female followers, “Liu Genghong Girls.” Similarly, German fitness icon Pamela Reif has attracted over 10.16 million fans through her livestream workout sessions (Chen et al., 2024). Essentially, these fitness influencers act as communicators, disseminating health and fitness information while offering free access to fitness resources. From a media communication perspective, the media characteristics of fitness videos—particularly perceived usefulness (PU) and perceived ease of use (PEOU)—critically shape individuals’ motivation to exercise (Liu and Shi, 2022). At the same time, the characteristics of celebrity figures also play a crucial role in communication. High-profile fitness influencers draw attention through their attractiveness, expertise, and trustworthiness, thereby enhancing viewers’ credibility perceptions and motivation to exercise (Spry et al., 2011). Moreover, the real-time interaction and encouragement between live streamers and viewers fosters a sense of virtual presence, stimulating viewers’ self-awareness and generating a strong social presence (SP). This process often leads to a sense of identification with the live streamer, ultimately strengthening behavioral intention (Yu et al., 2025). Gender significantly influences health and physical activity (Bidmon and Terlutter, 2015). Gender motivations for exercise differ: women are more motivated by goals related to weight management or physical appearance, while men are more likely to pursue endurance or strength training (Azevedo et al., 2007; González-Cutre et al., 2011). Accordingly, fitness livestreaming may be particularly effective in enhancing women’s willingness to exercise and improving their health status, offering a new and inclusive pathway for promoting national fitness.

Currently, research on live streaming’s influence on user behavior has developed in multiple directions, focusing primarily on areas such as consumer purchase intention (Chi et al., 2025), participation in tourism consumption (Ye et al., 2022), internet addiction (Jianfeng et al., 2024), and exercise behavior during the COVID-19 pandemic (Kim, 2022). Still, the effect of fitness streaming on female workout motivation post-pandemic lacks robust scholarly investigation. Moreover, the synergistic mechanisms between live-streaming celebrities’ characteristics and media attributes in shaping exercise behavioral intention have not yet been fully explored. In response, this study adopts the Stimulus-Organism-Response (S-O-R) theory and focuses on female audiences to examine how celebrity Characteristics and media content features jointly influence women’s exercise behavioral intentions (EBI). In addition, this research investigates the moderating role of body satisfaction (BS), aiming to enrich the theoretical understanding of the motivational factors behind women’s exercise behavior in livestreaming contexts. Ultimately, the study seeks to provide practical insights for promoting a culture of national fitness through live-streamed fitness activities and supporting the broader dissemination of exercise behavior.

## 2 Theoretical framework and research hypotheses

### 2.1 Fitness live stream and stimulus–organism–response theory

The Stimulus–Organism–Response (S-O-R) theory, a classical behavioral model, has been widely used to explore individuals’ complex responses to environmental stimuli. To better understand how fitness livestreaming influences users’ EBI, this study adopts the S-O-R theoretical framework to analyze how external stimuli in complex environments affect behavioral responses through internal states (Mehrabian and Russell, 1974). In this framework, stimuli (S) are categorized into direct stimuli (e.g., sound, light, video) and symbolic stimuli (e.g., live streaming content, cultural symbols), both of which serve as the initial triggers for behavioral responses. The organism (O) represents the individual receiving the stimuli, who processes the external inputs through emotional and physiological states, translating them into internal psychological representations that influence subsequent behavioral responses. The response (R) refers to the observable or latent behavioral outcomes resulting from the interaction between stimulus and organism, including both explicit behaviors and implicit changes (such as attitudinal shifts and physiological responses) (Bagozzi, 1996; Mehrabian and Russell, 1974).

The S-O-R theory, originally introduced within the domain of environmental psychology, is now considered a foundational theory in modern cognitive psychology and has been widely applied in fields such as online learning (Zhai et al., 2023), social media behavior (Masrom et al., 2021), and fitness product purchase behaviors (Chiu et al., 2022), as well as in broader studies of online behavior and health. Recent studies grounded in the S-O-R model have demonstrated that short videos, as environmental stimuli, can significantly influence individuals’ internal states and overt behaviors. However, relatively few academic efforts have focused on applying this framework to the context of fitness livestreaming. Li extended the applicability of the S-O-R model to livestreaming contexts, illustrating how video attractiveness and flow experience impact consumer traffic and purchase intentions (Li et al., 2025). Likewise, when it comes to live-streamed fitness content, users are also influenced by external environmental stimuli, which can alter emotional states and ultimately shape behavioral intentions. Thus, the S-O-R framework underpins this research.

Based on this framework, celebrity characteristics—including expertise, trustworthiness, and attractiveness—as well as media content characteristics, such as PU and PEOU, are conceptualized as stimuli (S). The organism (O) refers to the user’s internal psychological state during interactive fitness livestreams, specifically SP. The response (R) is defined as the user’s intention to exercise with or in preparation for exercising with the fitness live streamer.

### 2.2 Celebrity characteristics and social presence

According to the Source Attractiveness Model and Source Credibility Theory, celebrities as information sources can attract attention and generate persuasive effects (Petty et al., 1997). The source credibility and valence model in social psychology posits that celebrity sources influence users’ persuasion outcomes through three key dimensions: expertise, trustworthiness, and attractiveness, thereby enhancing users’ psychological identification (Durau et al., 2022). Compared with ordinary fitness streamers, celebrity fitness influencers such as Liu Geng Hong and Pamela Reif exhibit significant advantages. On one hand, they possess systematic fitness knowledge and professional credentials, which showcase higher levels of attractiveness and expertise. On the other hand, the symbolic capital and social influence endowed by their celebrity status effectively increase users’ trust (Yang et al., 2024). As a result, celebrity characteristics imbue livestream content with both informational depth and emotional warmth, significantly enhancing female users’ perceived value by reducing psychological distance (Yuan, 2023).

In social psychology, SP denotes the extent to which users perceive a sense of “realness” during mediated communication (Ming et al., 2021). High SP is often characterized by social warmth and human-like interaction (Li and Songhe, 2024). Through strong professional knowledge and timely feedback, celebrity fitness influencers are able to meet the diverse needs of female users and offer professional advice (Gao et al., 2023; Zheng and Fu, 2024). These positive interactions foster a sense of being valued and cared for, which in turn enhances users’ SP. At the same time, the enthusiasm, expertise, and trustworthiness demonstrated by celebrity streamers help users become more engaged in the livestreaming experience, eliciting positive and enjoyable emotional responses (Liu and Shi, 2022), and creating a sense of “realness” similar to an authentic feeling akin to in-person interactions (Zhang et al., 2025). During this process, female users are likely to form strong parasocial relationships with the celebrity streamers, perceiving them as close friends, which in turn increases their motivation to engage in physical activity (Casaló et al., 2020). According to SP theory, the authoritative empowerment derived from a celebrity influencer’s expertise, combined with emotional connections rooted in trustworthiness and attractiveness, fosters both cognitive dependence and emotional trust among users, thereby enhancing SP (Chen and Liao, 2022).

Therefore, in the context of celebrity fitness live streams, the following hypotheses are proposed:

*H1a:* The expertise of celebrity characteristics has a significant positive effect on SP.

*H1b:* The trustworthiness of celebrity characteristics has a significant positive effect on SP.

*H1c:* The attractiveness of celebrity characteristics has a significant positive effect on SP.

### 2.3 Media content characteristics and social presence

Media content characteristics primarily refer to any valuable information presented in live-streamed content (Zerfass et al., 2016). Drawing on the Technology Acceptance Model (TAM), media content characteristics are key predictors of users’ acceptance of information technology, particularly in terms of PU and PEOU (Feng et al., 2021). PU captures how much a user thinks a particular technology or tool will improve their efficiency or effectiveness in completing tasks. On the other hand, PEOU measures how straightforward and effortless the technology feels to operate (Marangunić and Granić, 2015). The adoption of technology can significantly impact users’ sense of SP (Gefen and Straub, 1997).

All information transmission in fitness live streams is realized through online platforms. Modern live streaming platforms support easy access via mobile devices such as smartphones and tablets, offering user-friendly interfaces, flexible interaction modes, and time efficiency. These features make it possible to meet the needs of users at different stages, thereby improving overall satisfaction. Specifically, such platforms convey SP through streamlined modes of communication. The content features of live streaming allow products and services to be showcased more accurately and comprehensively. These dynamic presentations of useful information not only provide visual stimulation but also evoke associations and strengthen SP (Liu and Shi, 2022). Research indicates that fitness streamers who answer user queries in real-time via bullet comments and provide immediate feedback are more likely to establish positive interaction with viewers, reduce the perceived mediation, and enhance SP (Han et al., 2015; Mehrabian, 1967). Based on social interaction theory, interaction is considered a basic human need (Baumeister and Leary, 2017). Interactions in live stream chats—both between viewer to viewer and streamers and viewers—can foster a sense of virtual social belonging, thereby enhancing SP (Zhou et al., 2019). Moreover, livestreamers’ use of voice, body movements, and facial expressions enhances the quality of the fitness experience and reinforces users’ PU. The complementarity of multiple information channels and the synergy of multi-sensory inputs generate effective stimuli, allowing users to become immersed in the virtual live-streaming environment and strengthening SP (Peng et al., 2024).

Therefore, in the context of celebrity fitness live streams, this study proposes the following hypotheses:

*H2a:* The PU of media content characteristics has a significant positive impact on SP.

*H2b:* The PEOU of media content characteristics has a significant positive impact on SP.

### 2.4 The impact of social presence on exercise behavioral intention

Given the complexity and uncertainty inherent in individual behavior, behavioral intention is regarded as a key theoretical construct for predicting personal behavior (Baker and Crompton, 2000). Exercise Behavioral Intention (EBI) refers to the extent to which an individual is willing or subjectively likely to engage in exercise behavior while watching fitness livestreams (Ajzen and Driver, 1991). SP is a highly immersive psychological state that generates a sense of “reality” for users, positively influencing their attitudes and ultimately promoting the formation of corresponding behavioral intentions (Kreijns et al., 2022).

Previous research suggests that SP can bridge the psychological gap between users and livestreamers, enhancing trust in livestream content and encouraging behavioral intentions (Li et al., 2024). Moreover, SP can positively predict future user engagement intentions and behavioral indicators of participation (Andel et al., 2020). A study based on the S-O-R model found that in virtual workout environments, real-time interaction between streamers and users enhances SP and increases users’ willingness to engage in exercise (Hou et al., 2023). In the domain of sports and physical activity, a significant positive correlation exists between SP and EBI; the authenticity and vividness of fitness videos are more likely to motivate users to make exercise-related decisions (Chen et al., 2024).

Based on this, the present study posits that in the context of celebrity fitness live streams, the stronger the SP experienced by female users while watching fitness content, the greater their intention to follow the celebrity streamer in engaging in exercise behavior.

Therefore, in the scenario of live celebrity fitness, it is hypothesized that.

*H3a:* SP mediates between the attractiveness of celebrity Characteristics and female EBI.

*H3b:* SP mediates between celebrity trait reliability and female EBI.

*H3c:* SP mediates between celebrity trait expertise and female EBI.

*H3d:* SP mediates between PU and female EBI for media content Characteristics.

*H3e:* SP mediates between PEOU and female EBI for media content Characteristics.

### 2.5 The moderating role of body satisfaction

As a core dimension of body image, BS primarily reflects individuals’ satisfaction with their appearance (Tiggemann and Mccourt, 2013). BS is a crucial psychological driver of exercise participation, and prior research has reveals that exercise can effective enhance BS (Shang et al., 2021). Research indicates a strong inverse relationship between BS and workout motivation, especially in females (Martin Ginis et al., 2003). According to social comparison theory, when female users watch fitness videos, the greater the perceived appearance discrepancy between themselves and the fitness demonstrator, the lower their BS tends to be (Sun et al., 2023). There is also a notable positive correlation between exercise frequency and body dissatisfaction among women, suggesting that increased body surveillance triggered by exercise may reinforce negative self-evaluation (Tiggemann and Williamson, 2000). However, when individuals hold positive expectations toward their body image (e.g., striving for a slim figure) and perceive a feasible path toward achieving that goal, BS may act as a motivator for engaging in physical activity. Thus, low BS may, paradoxically, enhance women’s exercise intention. Within fitness livestreaming, the dynamic visual displays of the live streamer (e.g., body movements, body shape) and interactive features such as on-screen comments (Lee et al., 2015) provide multisensory stimulation. This can elevate SP, immersing users in the virtual fitness environment and prompting them to evaluate their BS more critically. Specifically, high levels of perceived “co-presence” may strengthen the moderating influence of BS on the link between SP and EBI.

Thus, this research suggests that BS significantly influences the interplay between SP and EBI as a key mediator. When users have lower BS, SP exerts a stronger positive effect on EBI. In contrast, high BS may weaken this relationship due to the effects of downward or lateral comparison (Fardouly et al., 2021).

Thus, we hypothesize:

*H4:* BS negatively moderates the relationship between SP and EBI in the context of celebrity fitness livestreams.

### 2.6 Hypotheses and conceptual model

**Figure 1:**
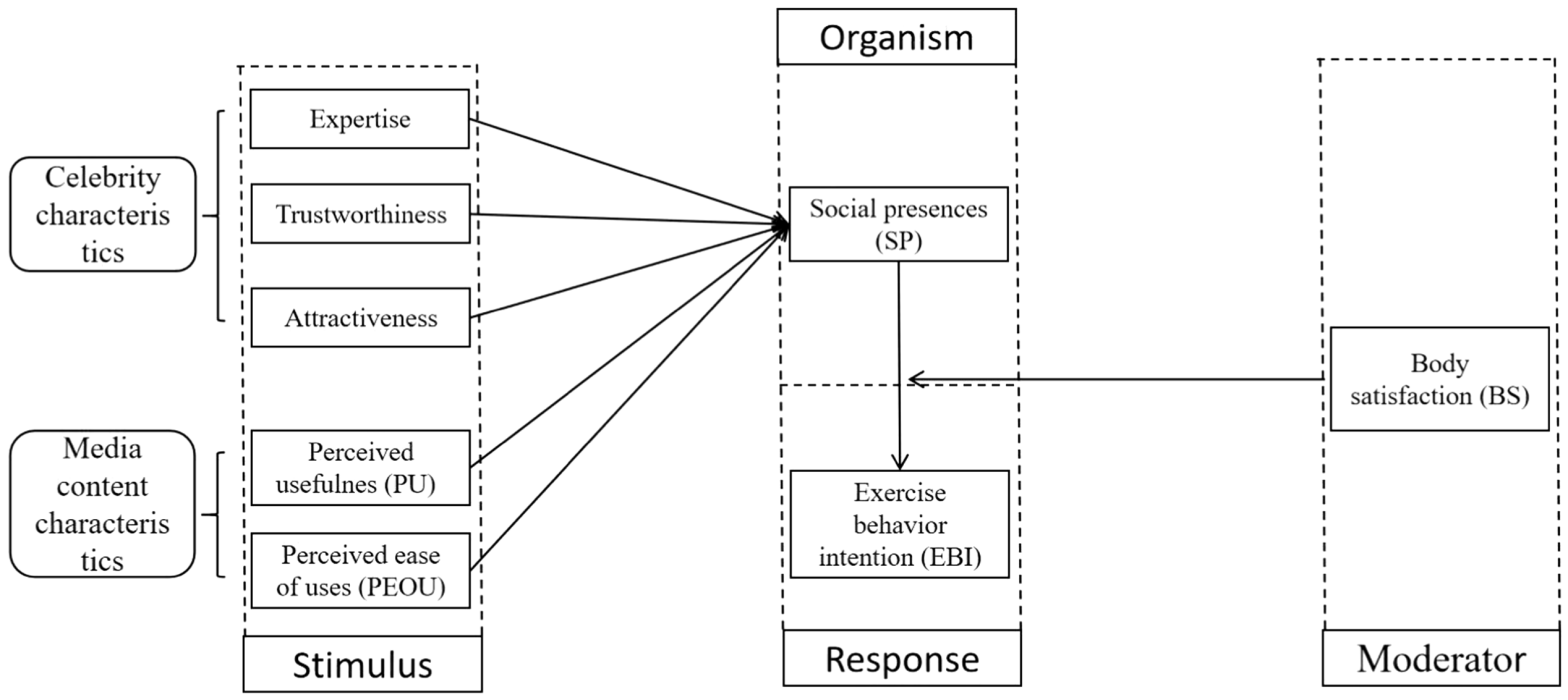
Hypothetical model.

Based on the above literature review and hypotheses, this study constructs a research model in which celebrity characteristics and media content characteristics serve as independent variables, EBI as the dependent variable, SP as a mediating variable, and BS as a moderating variable. As shown in Figure 1, the model outlines the relationships among celebrity characteristics, media content characteristics, SP, BS, and EBI. SP mediates the effect of celebrity characteristics and media content characteristics on EBI, while BS moderates the relationship between SP and EBI.

## 3 Methods

### 3.1 Participants

To explore the mechanism through which celebrity fitness livestreams influence women’s EBI, this study developed an online questionnaire using the Wenjuanxing (WJX) platform in December 2024. The questionnaire was distributed within fan communities of fitness celebrities, and 50 valid responses were initially collected for a pilot analysis. Results indicated good reliability and validity, and feedback was used to refine the questionnaire in terms of clarity, readability, and explanation of specific terms. Subsequently, from February to March 2025, a convenience sampling method was employed to distribute the final questionnaire within fan groups of celebrities such as Liu Genghong and Pamela Reif, as well as broader fitness communities. A total of 423 questionnaires were randomly distributed, and 382 valid responses were collected. As shown in Table 1, among respondents, 39.01% were aged 25–35, 27.75% aged 35–44, 21.47% aged 18–25, and 11.78% aged 45–55.

**Table 1:** Demographic characteristics of the sample.

Name	Option	Frequency	Percentage (%)	Cumulative percentage (%)
Age	18–25 years	82	21.47	21.47
	25–35 years	149	39.01	60.47
	35–45 years	106	27.75	88.22
	45–55 years	45	11.78	100
Education	Middle school and Below	19	4.97	4.97
	High school	49	12.83	17.8
	Junior college	87	22.77	40.58
	Bachelor degree and higher	227	59.42	100
Level of health	Health	113	29.58	29.58
	Sub-health	178	46.6	76.18
	Disease	91	23.82	100
Marital status	Married	218	57.07	57.07
	Unmarried	97	25.39	82.46
	Divorced	51	13.35	95.81
	Widowed	16	4.19	100
Total		382	100	100

Inclusion criteria: (1) adult females; (2) provided informed consent and had no cognitive impairments.

Exclusion criteria: (1) duplicate IP addresses; (2) survey completion time under 2 min; (3) responses that were patterned or exhibited a high degree of consistency were identified. As a result of this screening procedure, 18 questionnaires were excluded from the final analysis.

All participants provided informed consent. The study protocol was approved by the Ethics Committee of Chengdu Sport University (Approval Code: 2023-108).

All participants in this survey were informed, and the study was approved and cleared by the Ethics Committee of Chengdu sport university, Code: 2023-108.

### 3.2 Control variables

Since women’s EBI is influenced by individual and social factors, age, education, health level, and marital status were used as control variables in this study to reduce statistical bias.

### 3.3 Measurements

#### 3.3.1 Celebrity characteristics

This scale was developed based on the source credibility models by Ohanian (1990), adapted to Chinese female audiences and language contexts. It has been widely used in livestreaming research (Belanche et al., 2021). The scale consists of 12 items across three dimensions (expertise, trustworthiness, and attractiveness) rated on a 5-point Likert scale. Higher scores indicate stronger perceived celebrity characteristics. The Cronbach’s coefficient for this scale was 0.867.

#### 3.3.2 Media content characteristics

This scale was developed based on the TAM (Davis, 1989), assessing PU and PEOU. It includes six items rated on a 5-point Likert scale. Higher scores indicate higher perceived content quality. Cronbach *α* was 0.823.

#### 3.3.3 Social presence

SP was measured using a livestreaming-specific scale developed by Ying et al. (2021), which consists of 10 items across three dimensions: co-presence, communicative presence, and emotional presence. It is scored on a 5-point Likert scale, with a maximum total of 50 points. Higher scores indicate stronger perceived SP. Cronbach *α* was 0.867.

#### 3.3.4 Body satisfaction

BS was assessed using a modified version of the body image scale developed by Cash et al. (2002), adapted for this study’s context. This 6-item scale, rated on a 5-point Likert scale, has been widely used in BS research (Engeln et al., 2020). As BS is treated as a negative variable in this study, reverse scoring was applied. Higher scores indicate lower BS. Cronbach *α* was 0.910.

#### 3.3.5 Exercise behavior intention

EBI was measured using a scale adapted from Ajzen’s theory of planned behavior (Ajzen and Driver, 1991; Ajzen and Madden, 1986), which has been widely used in studies of behavioral attitudes. It contains three items on a 5-point Likert scale. Higher scores indicate stronger EBI. Cronbach *α* was 0.800.

#### 3.3.6 Statistical analysis

First, SPSS 26.0 was used to assess the reliability and validity of the measurement scales. AMOS 24.0 was then used to perform confirmatory factor analysis (CFA) and model fitting. Harman’s single-factor test was employed to evaluate common method bias. Results showed that the first factor explained only 33.335% of the variance, below the 40% threshold, indicating no serious common method bias.

Pearson correlation analysis was conducted to examine the relationships between variables such as attractiveness, trustworthiness, expertise, PU, PEOU, SP, BS, and EBI. Structural equation modeling was used to validate the role of SP mediation and to assess the fit of the model.

Finally, linear regression was used to examine the moderating role of BS in the relationship between SP and EBI. A simple slope analysis was conducted to visualize the moderating effect.

## 4 Results

### 4.1 Reliability and validity analysis

Confirmatory factor analysis (CFA) is a key approach for testing the validity of model constructs (Thompson and Daniel, 1996). As shown in Table 2, the composite reliability (CR) values of all constructs range from 0.759 to 0.911, and the average variance extracted (AVE) values range from 0.512 to 0.655. All AVE values exceed the 0.5 threshold, and CR values are greater than 0.7, indicating good reliability and convergent validity (Chin, 1998). Furthermore, the goodness-of-fit indices of the CFA results in Table 3 show that the model fits well. Finally, for discriminant validity, as shown in Table 4, the square root of the AVE for each construct is greater than the absolute value of its correlations with other constructs, indicating satisfactory discriminant validity.

**Table 2:** Confirmatory factor analysis.

Dimension	Items	Parameters of significant test					SMC	CR	AVE
		Estimate	S.E.	C.R.	*p*	Std. Estimate			
SP	SP1	1				0.712	0.516	0.759	0.512
	SP2	1.078	0.123	8.739	***	0.717	0.514		
	SP3	1.186	0.136	8.695	***	0.718	0.506		
Attractiveness	A1	1				0.821	0.673	0.814	0.593
	A2	0.827	0.058	14.337	***	0.738	0.544		
	A3	0.805	0.055	14.577	***	0.75	0.562		
Trustworthiness	T1	1				0.786	0.617	0.843	0.573
	T2	0.895	0.062	14.371	***	0.752	0.565		
	T3	0.973	0.068	14.288	***	0.748	0.559		
	T4	1.043	0.073	14.194	***	0.743	0.552		
Expertise	Exp1	1				0.768	0.589	0.852	0.590
	Exp2	0.955	0.065	14.717	***	0.773	0.597		
	Exp3	0.883	0.061	14.38	***	0.755	0.571		
	Exp4	0.902	0.061	14.797	***	0.777	0.604		
PU	PU1	1				0.786	0.618	0.810	0.588
	PU2	1.095	0.076	14.346	***	0.802	0.643		
	PU3	0.866	0.066	13.062	***	0.709	0.503		
PEOU	PEOU1	1				0.815	0.664	0.850	0.655
	PEOU2	0.844	0.057	14.922	***	0.728	0.53		
	PEOU3	1.122	0.063	17.896	***	0.878	0.772		
BS	BS1	1				0.787	0.62	0.911	0.629
	BS2	0.992	0.059	16.804	***	0.796	0.634		
	BS3	1.009	0.061	16.659	***	0.791	0.625		
	BS4	1.034	0.061	17.006	***	0.804	0.646		
	BS5	0.911	0.055	16.424	***	0.782	0.611		
	BS6	1.033	0.061	16.906	***	0.8	0.64		
EBI	EBI1	1				0.76	0.578	0.802	0.575
	EBI2	1.17	0.078	14.934	***	0.801	0.641		
	EBI3	1.028	0.077	13.312	***	0.711	0.506		

***p < 0.001.

**Table 3:** Model fit indicators.

	*χ*^2^/df	*p*	GFI	SRMR	CFI	NFI	IFI	RMSEA
Model	1.223	0.000	0.912	0.040	0.982	0.911	0.982	0.024

**Table 4:** Distinguishing validity: Pearson’s correlation vs. AVE square root values.

	Attractiveness	Trustworthiness	Expertise	PU	PEOU	BS	EBI	SP
Attractiveness	**0.77**							
Trustworthiness	0.525***	**0.757**						
Expertise	0.545***	0.450***	**0.768**					
PU	0.539***	0.404***	0.446***	**0.767**				
PEOU	0.608***	0.411***	0.520***	0.525***	**0.81**			
BS	−0.492***	−0.412***	−0.375***	−0.456***	−0.441***	**0.793**		
EBI	0.648***	0.535***	0.578***	0.591***	0.626***	−0.692***	**0.758**	
SP	0.668***	0.554***	0.599***	0.611***	0.632***	−0.469***	0.692***	**0.716**

Bolded black numbers on the diagonal are AVE square root values. ***p < 0.001.

### 4.2 Correlation analysis among variables

Pearson correlation analysis was conducted to examine the linear relationships and statistical significance between continuous variables. As shown in Table 5, the correlation coefficients and significance levels are as follows: Attractiveness and EBI (*r* = 0.523, *p* < 0.01), Trustworthiness and EBI (*r* = 0.445, *p* < 0.01), Expertise and EBI (*r* = 0.478, *p* < 0.01), PU and EBI (*r* = 0.470, *p* < 0.01), PEOU and EBI (*r* = 0.516, *p* < 0.01), SP and EBI (*r* = 0.514, *p* < 0.01), BS and EBI (*r* = −0.595 *p* < 0.01). All correlation coefficients between Attractiveness, Trustworthiness, Expertise, PU, PEOU, and EBI are positive, indicating a positive relationship. In contrast, the correlation between BS and EBI is negative, suggesting a negative relationship.

**Table 5:** Pearson correlation test.

	Attractiveness	Trustworthiness	Expertise	PU	PEOU	SP	BS	EBI
Attractiveness	1							
Trustworthiness	0.440**	1						
Expertise	0.447**	0.388**	1					
PU	0.434**	0.338**	0.369**	1				
PEOU	0.500**	0.344**	0.439**	0.425**	1			
SP	0.498**	0.429**	0.460**	0.455**	0.487**	1		
BS	−0.423**	−0.365**	−0.333**	−0.394**	−0.391**	−0.374**	1	
EBI	0.523**	0.445**	0.478**	0.470**	0.516**	0.514**	−0.595**	1

*p < 0.05; **p < 0.01.

### 4.3 Common method bias test

In this study, Harman’s one-factor test was used to assess common method bias. An exploratory factor analysis showed that nine factors had eigenvalues greater than 1. The first factor accounted for 32.853% of the variance, which is below the 40% threshold. Thus, the potential for common method bias to distort the research findings appears negligible in the current study.

### 4.4 Hypothesis path coefficient testing

Structural equation modeling (SEM) was employed to examine the causal relationships and mediating effects among the variables. A path is considered statistically significant if *p* < 0.05, and a positive coefficient indicates a significant positive effect of the independent variable on the dependent variable. As shown in Table 6, Attractiveness has a positive effect on SP (*β* = 0.128, SE = 0.038), supporting H1c. Trustworthiness positively influences SP (*β* = 0.115, SE = 0.034), supporting H1b. Expertise has a significant positive effect on SP (*β* = 0.117, SE = 0.035), supporting H1a. PU positively affects SP (*β* = 0.142, SE = 0.036), supporting H2a. PEOU also shows a positive impact on SP (*β* = 0.137, SE = 0.037), supporting H2b. SP significantly and positively influences EBI (*β* = 1.265, SE = 0.141), supporting H3.

**Table 6:** Model path regression results.

Path			Estimate	S.E.	C.R.	*p*	Std. Estimate
SP	←	Attractiveness	0.128	0.038	3.362	***	0.251
SP	←	Trustworthiness	0.115	0.034	3.344	***	0.195
SP	←	Expertise	0.117	0.035	3.374	***	0.207
SP	←	PU	0.142	0.036	3.962	***	0.253
SP	←	PEOU	0.137	0.037	3.676	***	0.245
EBI	←	SP	1.265	0.141	8.958	***	0.823

*p < 0.05; **p < 0.01; ***p < 0.001.

### 4.5 Mediation analysis in SEM

**Figure 2:**
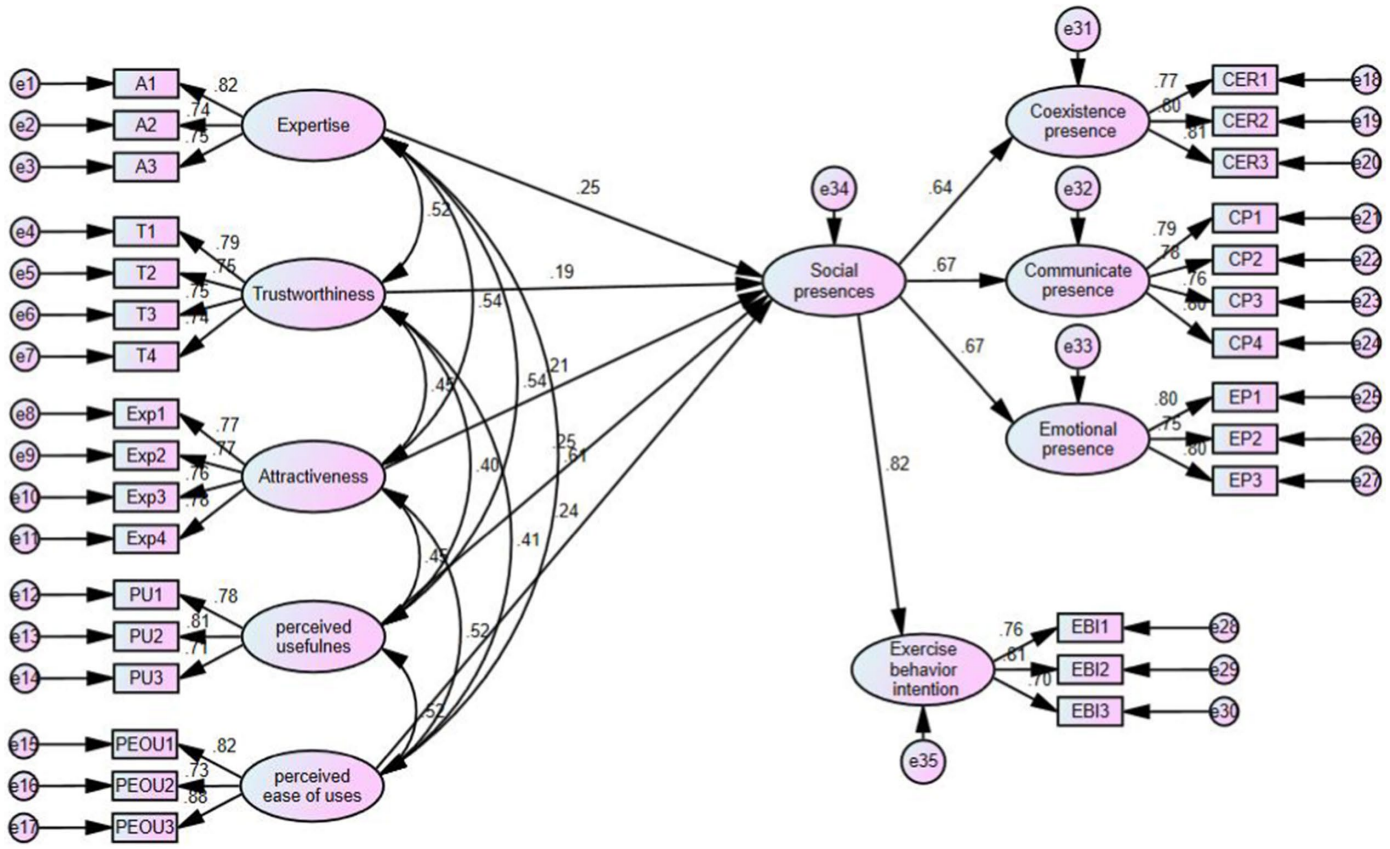
Visualization of intermediary effects.

Figure 2 presents the standardized path coefficients model assessing the mediating effects of SP on the relationships between fitness short video characteristics and EBI. According to the model fit indices in Table 7, the mediation model for Attractiveness, Trustworthiness, Expertise, PU, and PEOU demonstrates a good fit: *χ*^2^/df < 3, GFI > 0.9, SRMR < 0.05, CFI > 0.9, RMSEA < 0.05, and NFI > 0.9.

**Table 7:** Model fit indicators.

	*χ*^2^/df	*p*	GFI	SRMR	CFI	NFI	IFI	RMSEA
Model	1.272	0.000	0.923	0.044	0.981	0.918	0.981	0.027

As shown in Figure 2, the path coefficients for Attractiveness → SP (*β* = 0.21), Trustworthiness → SP (*β* = 0.19), Expertise → SP (*β* = 0.25), and SP → EBI (*β* = 0.82) are all significant, indicating that SP mediates the relationship between celebrity characteristics and EBI. Hence, H3a, H3b, and H3c are supported. Similarly, the significant path coefficients of PU → SP (*β* = 0.25), PEOU → SP (*β* = 0.24), and SP → EBI (*β* = 0.82) indicate that SP also mediates the relationship between media content characteristics and EBI, supporting H3d and H3e.

This study used AMOS 24.0 and bootstrapping with 5,000 resamples to test the mediating effects, with Attractiveness, Trustworthiness, Expertise, PU, and PEOU as independent variables, EBI as the dependent variable, and SP as the mediator. As shown in Table 8, all mediation paths were statistically significant, as the 95% confidence intervals did not include zero. The five mediation paths—Attractiveness → SP → EBI, Trustworthiness → SP → EBI, Expertise → SP → EBI, PU → SP → EBI, and PEOU → SP → EBI—are all supported, confirming that SP plays a mediating role in the relationships between celebrity/media content Characteristics and EBI.

**Table 8:** Mediation effect test.

Path	Estimate	Lower	Upper	*p*
Attractiveness → SP → EBI	0.162	0.061	0.282	0.002
Trustworthiness → SP → EBI	0.145	0.061	0.249	0.001
Expertise → SP → EBI	0.149	0.058	0.252	0.001
PU → SP → EBI	0.18	0.085	0.296	0.000
PEOU → SP → EBI	0.173	0.076	0.283	0.001

### 4.6 Moderation analysis

Hierarchical regression analysis was conducted to examine the moderating role of BS. As shown in Table 9, Model 1 examines the effect of the independent variable SP on the dependent variable EBI without considering the moderator. In Model 3, both SP and BS were mean-centered, and their interaction term (SP × BS) was included in the regression model. The regression coefficient for the interaction term was (*β* = −0.115, *p* < 0.05), indicating a significant negative moderating effect of BS on the relationship between SP and EBI.

**Table 9:** Results of moderated effects analysis (*n* = 382).

	M1			M2			M3		
	*β*	*t*	*p*	*β*	*t*	*p*	*β*	*t*	*p*
PE	0.514	11.668	0.000**	0.338	8.257	0.000**	0.37	8.818	0.000**
BS				−0.469	−11.448	0.000**	−0.453	−11.062	0.000**
PE*BS							−0.115	−2.952	0.003**
*R* ^2^	0.264			0.453			0.465		
Adjusted *R*^2^	0.262			0.45			0.461		
*F*	*F* = 136.141, *p* = 0.000			*F* = 156.900, *p* = 0.000			*F* = 109.633, *p* = 0.000		

Dependent variable: EBI.

*p < 0.05; **p < 0.01.

**Figure 3:**
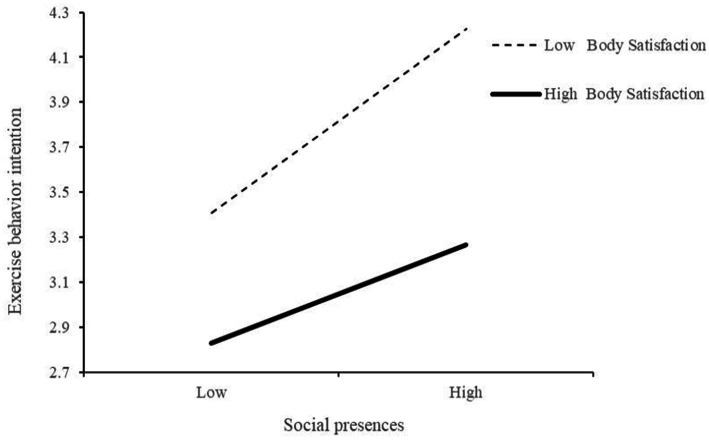
Mediation diagram of BS between SP and EBI.

To further explore this moderating effect, Figure 3 illustrates that the positive predictive effect of SP on EBI is stronger for individuals with low BS (−1 SD; simple *β* = 0.590, *p* < 0.001) than for those with high BS (+1 SD; simple *β* = 0.317, *p* < 0.001). In other words, the positive impact of SP on EBI diminishes as BS, increases, suggesting that women with lower levels of BS are more strongly influenced by SP in terms of exercise behavioral intention.

## 5 Discussion

### 5.1 The impact of celebrity characteristics on women’s exercise behavioral intention

The results of this study indicate that the three core Characteristics of celebrity figures—expertise, trustworthiness, and attractiveness—positively predict women’s exercise behavioral intention EBI through the enhancement of SP. This finding aligns with prior research, suggesting that celebrity Characteristics can boost SP, which in turn influences behavioral intention (Huang et al., 2023). Regarding expertise, live streamers’ demonstration of technical proficiency, professional knowledge, and skilled interaction can increase user interest and SP (Li et al., 2022). For women with clear fitness goals, celebrity live streamers’ rich knowledge of exercise significantly enhances user focus and immersion, thereby strengthening the effects of SP on EBI.

From the perspective of trustworthiness, source credibility theory posits that the public image and social influence of celebrity live streamers create dual anchors of trust. This symbolic identity reduces users’ information-filtering costs and enhances feelings of security and trust (Djafarova and Rushworth, 2017). Moreover, real-time interaction through “bullet comments” (danmu) fosters a sense of social engagement, which deepens the mediating role of SP in promoting EBI (Cheng et al., 2024).

As for attractiveness, the source attractiveness model (Aw and Chuah, 2021) suggests that the livestreamers’ physical appeal and charm can have a visual impact on female viewers. This not only increases positive feelings toward the streamer but also associates fitness behaviors with positive aesthetic experiences, drawing users into the virtual fitness space and generating SP (Changzhen and Xinyi, 2023), which ultimately stimulates their intention to follow along.

In sum, celebrity fitness livestreamers can positively influence users’ behavioral intentions (Chen et al., 2024). According to social identity theory, individuals derive self-worth through group membership. The stronger the group identity, the more likely individuals are to internalize group norms (Hogg, 2016). For instance, cultural symbols such as “Liu Genghong Girls” or “Pamela Girls” reflect processes of symbolic interaction and identity reconstruction within virtual fitness communities. Celebrity livestreamers construct virtual social spaces that foster quasi-social relationships with users, mitigating feelings of loneliness and disconnection in fast-paced societies. Motivated by the need for group belonging, users participate in collective fitness rituals. Moreover, celebrities as opinion leaders can enhance user trust, significantly increasing SP and positively influencing women’s EBI (Casaló et al., 2020).

### 5.2 The impact of media content characteristics on exercise behavioral intention

This study finds that PU and PEOU of media content significantly predict women’s EBI through SP. These findings are consistent with studies in online learning contexts (Humida et al., 2022). Based on the TAM and SP theory, both PU and PEOU jointly influence SP, which in turn affects women’s exercise intentions.

Regarding PEOU, live streaming platforms such as Douyin, Kuaishou, and Bilibili have optimized their interface design through features like one-click access, quick comment functions, and real-time feedback, which reduce operational complexity and lower the participation threshold. This “cognitive resource liberation” enhances SP. Additionally, the use of catchy music and motivational slogans helps concentrate users’ attention and trigger emotional resonance, further motivating female users to engage in exercise (Tide, 2022).

In terms of content construction, many live streams adopt home-based, conversational formats with simple, repetitive movements. This lifestyle-oriented presentation allows users to shift from “learning technology” to “enjoying social interaction,” thereby deepening SP and enhancing EBI (Hou et al., 2023).

In terms of PU, live-streamed workouts help women achieve ideal body shapes and meet physical and mental health needs. The explicit functional value of this content increases users’ focus and immersion, thereby enhancing SP and stimulating exercise intentions (Liu and Shi, 2022). Moreover, the integration of verbal and non-verbal cues (e.g., expressions, gestures, tone) with interactive features like comments offers users multi-sensory stimulation. These combined information channels help users form a clear understanding of the utility of exercise, strengthening the impact of SP (Xiang et al., 2016).

Flow theory further explains the synergy between PU and PEOU. High levels of both contribute to immersive experiences that amplify SP and consequently enhance EBI (Harris et al., 2023). According to TAM, the evolution of women’s EBI results from the cognitive relief offered by PEOU and the value promise driven by PU (Karahanna and Straub, 1999). PEOU is particularly critical for first-time users, forming the foundation for PU. Over time, PU becomes the core determinant of continued use, acting as an anchor for sustained behavioral intention. Therefore, national fitness initiatives should leverage the dual effect of PEOU and PU—reducing initial barriers while enhancing long-term engagement through data-driven optimization and social relationship building.

### 5.3 The moderating role of body satisfaction on SP and EBI

This study finds that the effect of SP on EBI is moderated by BS, with a stronger influence observed among women with lower BS. This finding is consistent with prior studies (Ginis et al., 2008; Martin Ginis et al., 2003). Specifically, the relationship between SP and EBI is more significant when BS is low (*β* = −0.115, *p* = 0.003 < 0.01), suggesting that low BS strengthens the influence of SP on EBI.

According to social comparison theory (Festinger, 1954), individuals naturally evaluate themselves by comparing to others. When women with low BS compare themselves upwardly to ideal body types portrayed by fitness celebrities, the perceived gap may trigger a motivation for self-improvement (Tiggemann and Polivy, 2010). Watching fitness livestreams may heighten awareness of this gap, thereby increasing motivation to exercise (Ginis et al., 2008).

Furthermore, sociocultural theory posits that media transmit cultural pressures and internalized beauty standards. In response, individuals may adopt cost-effective and accessible exercise methods to enhance BS (Thompson et al., 1999). Thus, lower BS may increase women’s motivation to engage in fitness activities, strengthening the impact of SP on EBI. Conversely, higher BS may weaken this relationship.

### 5.4 Theoretical implications

This study makes several theoretical contributions. First, it investigates the impact of fitness celebrity livestreaming on women’s EBI in the post-pandemic era, shedding light on the complex mechanisms behind exercise intention and enriching the literature on physical activity and health promotion. Second, the study extends and validates the S-O-R model, offering a comprehensive framework for understanding women’s exercise behavior. Third, it demonstrates the moderating role of BS, contributing to the literature on body image and highlighting the relevance of BS in the context of exercise behavior. This provides a new perspective on promoting physical activity among women. Lastly, by examining the joint effects of celebrity Characteristics and media content features, the study advances theoretical understanding of how these factors influence EBI, enriching both the TAM and source credibility theory, and offering new strategies for promoting online fitness engagement.

### 5.5 Practical implications

This study offers four key practical implications. First, under the S-O-R model, the positive impact of PU and PEOU on EBI highlights the importance of live streaming technology’s convenience and perceived value. Given the accessibility, companionship, and cost-effectiveness of livestream fitness, it is likely to become a widely adopted method for national fitness. The inclusivity and diversity of live-streamed content can meet varying user needs and improve exercise adherence.

Second, the mediating role of SP offers new insights for fitness promotion. Enhancing SP in live streaming can boost users’ exercise motivation. Thus, livestreamers and public health agencies should leverage this factor to create immersive fitness experiences.

Third, the importance of celebrity Characteristics in influencing women’s exercise intentions suggests that fitness influencers can enhance engagement through more appealing content. Governments and platforms should cultivate diverse fitness figures to meet the needs of different populations.

Lastly, the moderating role of BS underscores the importance of individual psychological factors. Public campaigns and platform strategies should promote healthy body image ideals and create fitness programs tailored to body confidence and self-perception, thereby increasing motivation to exercise.

### 5.6 Limitations

This study has several limitations. First, as a cross-sectional study, it only captures associations at a single time point and cannot determine causal relationships. Future studies should incorporate control groups or adopt longitudinal designs. Second, the cross-sectional nature prevents tracking dynamic changes in variables over time, possibly missing transitional patterns. Retrospective studies could explore how BMI and BS vary across life stages. Thirdly, this study focuses on women who are concerned about live-streaming fitness. Future research could examine other demographic data, such as those of men, teenagers or the elderly, to assess its universality. Finally, although this study explored SP as a mediator and BS as a moderating factor, other variables (such as flow experience) may also affect the relationship between celebrity live streaming and female EBI, which requires further research in future studies.

## 6 Conclusion

This study, grounded in the S-O-R framework, validates a moderated mediation model that elucidates the mechanisms underlying the impact of fitness livestreaming on women’s EBI. The findings indicate that both celebrity characteristics (professionalism, reliability, attractiveness) and media content attributes (perceived usefulness, ease of use) significantly enhance women’s EBI by fostering a profound sense of SP. This confirms SP’s crucial mediating role in translating external stimuli into behavioral intentions. Furthermore, body BS was identified as a critical boundary condition that negatively moderates the relationship between SP and EBI; women with lower BS exhibited stronger behavioral responses, highlighting the motivational power of self-improvement drives.

Based on these theoretical insights, several specific recommendations are proposed: (1) Platforms should cultivate influencers who embody established traits of credibility and attractiveness to maximize social presence; (2) Content must be optimized for perceived usefulness and ease of use to reduce participation barriers; (3) Public health initiatives should leverage livestreaming to target individuals with lower body satisfaction, channeling their motivation for self-improvement into sustained exercise habits. This approach would effectively implement national fitness strategies.

## Data Availability

The original contributions presented in the study are included in the article/supplementary material, further inquiries can be directed to the corresponding author/s.

## References

[ref1] Ajzen I. Driver B. L. (1991). Prediction of leisure participation from behavioral, normative, and control beliefs: an application of the theory of planned behavior. Leis. Sci. 13, 185–204. doi: 10.1080/01490409109513137

[ref2] Ajzen I. Madden T. J. (1986). Prediction of goal-directed behavior: attitudes, intentions, and perceived behavioral control. J. Exp. Soc. Psychol. 22, 453–474. doi: 10.1016/0022-1031(86)90045-4

[ref3] Andel S. A. De Vreede T. Spector P. E. Padmanabhan B. Singh V. K. De Vreede G.-J. (2020). Do social features help in video-centric online learning platforms? A social presence perspective. Comput. Hum. Behav. 113:106505. doi: 10.1016/j.chb.2020.106505

[ref4] Aw E. C.-X. Chuah S. H.-W. (2021). “Stop the unattainable ideal for an ordinary me!” fostering parasocial relationships with social media influencers: the role of self-discrepancy. J. Bus. Res. 132, 146–157. doi: 10.1016/j.jbusres.2021.04.025

[ref5] Azevedo M. R. Araújo C. L. P. Reichert F. F. Siqueira F. V. Da Silva M. C. Hallal P. C. (2007). Gender differences in leisure-time physical activity. Int. J. Public Health 52, 8–15. doi: 10.1007/s00038-006-5062-1, PMID: 17966815 PMC2778720

[ref6] Bagozzi R. P. (1996). The role of arousal in the creation and control of the halo effect in attitude models. Psychol. Mark. 13, 235–264. doi: 10.1002/(SICI)1520-6793(199605)13:3<>3.0.CO;2-D

[ref7] Baker D. A. Crompton J. L. (2000). Quality, satisfaction and behavioral intentions. Ann. Tour. Res. 27, 785–804. doi: 10.1016/S0160-7383(99)00108-5

[ref8] Baker D. Mead N. Campbell S. (2002). Inequalities in morbidity and consulting behaviour for socially vulnerable groups. Br. J. Gen. Pract. 52, 124–130, PMID: 11885821 PMC1314218

[ref9] Baumeister R. F. Leary M. R. (2017). “The need to belong: desire for interpersonal attachments as a fundamental human motivation” in Interpersonal development, 57–89. 7777651

[ref10] Belanche D. Casaló L. V. Flavián M. Ibáñez-Sánchez S. (2021). Building influencers' credibility on Instagram: effects on followers’ attitudes and behavioral responses toward the influencer. J. Retail. Consum. Serv. 61:102585. doi: 10.1016/j.jretconser.2021.102585

[ref11] Bidmon S. Terlutter R. (2015). Gender differences in searching for health information on the internet and the virtual patient-physician relationship in Germany: exploratory results on how men and women differ and why. J. Med. Internet Res. 17:E156. doi: 10.2196/jmir.4127, PMID: 26099325 PMC4526954

[ref12] Bull F. C. Al-Ansari S. S. Biddle S. Borodulin K. Buman M. P. Cardon G. . (2020). World Health Organization 2020 guidelines on physical activity and sedentary behaviour. Br. J. Sports Med. 54, 1451–1462. doi: 10.1136/bjsports-2020-102955, PMID: 33239350 PMC7719906

[ref13] Casaló L. V. Flavián C. Ibáñez-Sánchez S. (2020). Influencers on Instagram: antecedents and consequences of opinion leadership. J. Bus. Res. 117, 510–519. doi: 10.1016/j.jbusres.2018.07.005

[ref14] Cash T. F. Fleming E. C. Alindogan J. Steadman L. Whitehead A. (2002). Beyond body image as a trait: the development and validation of the body image states scale. Eat. Disord. 10, 103–113. doi: 10.1080/10640260290081678, PMID: 16864251

[ref16] Changzhen H. Xinyi Y. (2023). The influence of information source characteristics of live broadcast anchors on consumers' brand attitudes. Bus. Econ. Res. 17, 68–73.

[ref17] Chen S. Hong J. Milton K. Klepac B. Ma J. Pedisic Z. (2023). Analysis of national physical activity and sedentary behaviour policies in China. BMC Public Health 23:1024. doi: 10.1186/s12889-023-15865-8, PMID: 37254122 PMC10230767

[ref18] Chen J. Liao J. (2022). Antecedents of viewers’ live streaming watching: a perspective of social presence theory. Front. Psychol. 13:839629. doi: 10.3389/fpsyg.2022.839629, PMID: 35432106 PMC9008234

[ref19] Chen X. Zhu Y. Xu X. (2024). Being there and being with them: the effects of visibility affordance of online short fitness video on users’ intention to cloud fitness. Front. Psychol. 15:1267502. doi: 10.3389/fpsyg.2024.1267502, PMID: 38362244 PMC10867133

[ref20] Cheng G. Li W. He M. Liao L. (2024). Exploring consumer responses to official endorsement: roles of credibility and attractiveness attributes in live streaming. Front. Psychol. 15:1371343. doi: 10.3389/fpsyg.2024.1371343, PMID: 38831950 PMC11146374

[ref21] Chi F. Wang D. Park S. Dai F. (2025). Decoding the viewer experience shaped by tourism live streaming: the pathway to commercial success. J. Travel Tourism Mark. 42, 65–84. doi: 10.1080/10548408.2024.2427163

[ref1002] Chin W. W. (1998). “The partial least squares approach to structural equation modeling,” in. Modern Methods for Business Research, (Psychology Press), 295–336.

[ref22] Chiu W. Oh G. E. Cho H. (2022). Impact of COVID-19 on consumers' impulse buying behavior of fitness products: a moderated mediation model. J. Consum. Behav. 21, 245–258. doi: 10.1002/cb.1998, PMID: 38607899 PMC8653049

[ref23] Davis F. D. (1989). Perceived usefulness, perceived ease of use, and user acceptance of information technology. MIS Q. 13, 319–340. doi: 10.2307/249008

[ref24] Djafarova E. Rushworth C. (2017). Exploring the credibility of online celebrities' Instagram profiles in influencing the purchase decisions of young female users. Comput. Hum. Behav. 68, 1–7. doi: 10.1016/j.chb.2016.11.009

[ref25] Durau J. Diehl S. Terlutter R. (2022). Motivate me to exercise with you: the effects of social media fitness influencers on users’ intentions to engage in physical activity and the role of user gender. Digital Health 8:205520762211027. doi: 10.1177/20552076221102769, PMID: 35615268 PMC9125114

[ref1001] Engeln R. Loach R. Imundo M. N. Zola A. (2020). Compared to Facebook, Instagram use causes more appearance comparison and lower body satisfaction in college women. Body image 34, 38–45. doi: 10.1016/j.bodyim.2020.04.007 32505866

[ref26] Fardouly J. Pinkus R. T. Vartanian L. R. (2021). Targets of comparison and body image in women’s everyday lives: the role of perceived attainability. Body Image 38, 219–229. doi: 10.1016/j.bodyim.2021.04.009, PMID: 33932884

[ref27] Feng G. C. Su X. Lin Z. He Y. Luo N. Zhang Y. (2021). Determinants of technology acceptance: two model-based meta-analytic reviews. J. Mass. Commun. Q. 98, 83–104. doi: 10.1177/1077699020952400

[ref28] Festinger L. (1954). A theory of social comparison processes. Hum. Relat. 7, 117–140. doi: 10.1177/001872675400700202

[ref29] Gao W. Jiang N. Guo Q. (2023). How do virtual streamers affect purchase intention in the live streaming context? A presence perspective. J. Retail. Consum. Serv. 73:103356. doi: 10.1016/j.jretconser.2023.103356

[ref30] Gefen D. Straub D. W. (1997). Gender differences in the perception and use of e-mail: an extension to the technology acceptance model. MIS Q. 21, 389–400. doi: 10.2307/249720

[ref31] Ginis K. A. M. Prapavessis H. Haase A. M. (2008). The effects of physique-salient and physique non-salient exercise videos on women's body image, self-presentational concerns, and exercise motivation. Body Image 5, 164–172. doi: 10.1016/j.bodyim.2007.11.005, PMID: 18486573

[ref32] González-Cutre D. Sicilia Á. Águila C. (2011). Interplay of different contextual motivations and their implications for exercise motivation. J. Sports Sci. Med. 10:274, PMID: 24149872 PMC3761864

[ref33] Han S. Min J. Lee H. (2015). Antecedents of social presence and gratification of social connection needs in SNS: a study of Twitter users and their mobile and non-mobile usage. Int. J. Inf. Manag. 35, 459–471. doi: 10.1016/j.ijinfomgt.2015.04.004

[ref34] Harris D. J. Allen K. L. Vine S. J. Wilson M. R. (2023). A systematic review and meta-analysis of the relationship between flow states and performance. Int. Rev. Sport Exerc. Psychol. 16, 693–721. doi: 10.1080/1750984X.2021.1929402

[ref1003] Hogg M. A. (2016). Social identity theory. in Understanding Peace and Conflict Through Social Identity Theory: Contemporary Global Perspectives(Cham: Springer International Publishing), 3–17.

[ref35] Hou J. Han B. Chen L. Zhang K. (2023). Feeling present matters: effects of social presence on live-streaming workout courses’ purchase intention. J. Prod. Brand. Manag. 32, 1082–1092. doi: 10.1108/JPBM-03-2022-3926

[ref36] Huang Z. Zhu Y. Hao A. Deng J. (2023). How social presence influences consumer purchase intention in live video commerce: the mediating role of immersive experience and the moderating role of positive emotions. J. Res. Interact. Mark. 17, 493–509. doi: 10.1108/JRIM-01-2022-0009

[ref37] Humida T. Al Mamun M. H. Keikhosrokiani P. (2022). Predicting behavioral intention to use e-learning system: a case-study in Begum Rokeya University, Rangpur, Bangladesh. Educ. Inf. Technol. 27, 2241–2265. doi: 10.1007/s10639-021-10707-9, PMID: 34413694 PMC8364304

[ref38] Jianfeng H. Xian Z. Zexiu A. (2024). Effects of physical exercise on adolescent short video addiction: a moderated mediation model. Heliyon 10:e29466. doi: 10.1016/j.heliyon.2024.e29466, PMID: 38638962 PMC11024624

[ref39] Karahanna E. Straub D. W. (1999). The psychological origins of perceived usefulness and ease-of-use. Inf. Manag. 35, 237–250. doi: 10.1016/S0378-7206(98)00096-2

[ref40] Kim M. (2022). How can I be as attractive as a fitness Youtuber in the era of COVID-19? The impact of digital attributes on flow experience, satisfaction, and behavioral intention. J. Retail. Consum. Serv. 64:102778. doi: 10.1016/j.jretconser.2021.102778

[ref41] Kimm S. Y. S. Glynn N. W. Mcmahon R. P. Voorhees C. C. Striegel-Moore R. H. Daniels S. R. (2006). Self-perceived barriers to activity participation among sedentary adolescent girls. Med. Sci. Sports Exerc. 38, 534–540. doi: 10.1249/01.mss.0000189316.71784.dc, PMID: 16540842

[ref42] Kreijns K. Xu K. Weidlich J. (2022). Social presence: conceptualization and measurement. Educ. Psychol. Rev. 34, 139–170. doi: 10.1007/s10648-021-09623-8, PMID: 34177204 PMC8217203

[ref43] Lee E. Lee J.-A. Moon J. H. Sung Y. (2015). Pictures speak louder than words: motivations for using Instagram. Cyberpsychol. Behav. Soc. Netw. 18, 552–556. doi: 10.1089/cyber.2015.0157, PMID: 26348817

[ref44] Li L. Kang K. Zhao A. Feng Y. (2022). The impact of social presence and facilitation factors on online consumers' impulse buying in live shopping–celebrity endorsement as a moderating factor. Inf. Technol. People 36, 2611–2631. doi: 10.1108/ITP-03-2021-0203

[ref45] Li P. Songhe X. (2024). How presence affects college students’ online learning outcomes: unveiling its underlying mechanism. Contemp. Soc. Sci. 17, 324–332.

[ref46] Li Z. Wang Y. Cianfrone B. A. Guo Z. Liu B. Zhang J. . (2025). Impact of scene features of E-commerce live streaming on consumers’ flow and purchase intentions of sporting goods. Behav. Sci. 15:238. doi: 10.3390/bs15020238, PMID: 40001869 PMC11851700

[ref47] Li N. Xuan C. Chen R. (2024). Different roles of two kinds of digital coexistence: the impact of social presence on consumers' purchase intention in the live streaming shopping context. J. Retail. Consum. Serv. 80:103890. doi: 10.1016/j.jretconser.2024.103890

[ref48] Liu L. Shi X. (2022). Influential mechanism of celebrity live broadcast fitness of audience’s fitness behavioral intention in view of Liu Genghong phenomenon. J. Wuhan Sports Univ. 56, 37–44.

[ref49] Marangunić N. Granić A. (2015). Technology acceptance model: a literature review from 1986 to 2013. Universal Access Inf. Soc. 14, 81–95. doi: 10.1007/s10209-014-0348-1

[ref50] Martin Ginis K. A. Jung M. E. Gauvin L. (2003). To see or not to see: effects of exercising in mirrored environments on sedentary women's feeling states and self-efficacy. Health Psychol. 22, 354–361. doi: 10.1037/0278-6133.22.4.354, PMID: 12940391

[ref51] Masrom M. B. Busalim A. Abuhassna H. Mahmood N. (2021). Understanding students’ behavior in online social networks: a systematic literature review. Int. J. Educ. Technol. Higher Educ. 18:6.

[ref52] Mehrabian A. (1967). Orientation behaviors and nonverbal attitude communication. J. Commun. 324–332. 5588696

[ref53] Mehrabian A. Russell J. A. (1974). An approach to environmental psychology. Paperback The MIT Press.

[ref54] Ming J. Jianqiu Z. Bilal M. Akram U. Fan M. (2021). How social presence influences impulse buying behavior in live streaming commerce? The role of SOR theory. Int. J. Web Inf. Syst. 17, 300–320. doi: 10.1108/IJWIS-02-2021-0012

[ref55] Ohanian R. (1990). Construction and validation of a scale to measure celebrity endorsers' perceived expertise, trustworthiness, and attractiveness. J. Advert. 19, 39–52. doi: 10.1080/00913367.1990.10673191

[ref56] Peng L. Zhang N. Huang L. (2024). How the source dynamism of streamers affects purchase intention in live streaming e-commerce: considering the moderating effect of Chinese consumers’ gender. J. Retail. Consum. Serv. 81:103949. doi: 10.1016/j.jretconser.2024.103949

[ref57] Petty R. E. Wegener D. T. Fabrigar L. R. (1997). Attitudes and attitude change. Annu. Rev. Psychol. 48, 609–647. doi: 10.1146/annurev.psych.48.1.609, PMID: 9046570

[ref58] Sallis R. E. (2009). Exercise is medicine and physicians need to prescribe it! Br. J. Sports Med. 43, 3–4. doi: 10.1136/bjsm.2008.054825, PMID: 18971243

[ref59] Shang Y. Xie H.-D. Yang S.-Y. (2021). The relationship between physical exercise and subjective well-being in college students: the mediating effect of body image and self-esteem. Front. Psychol. 12:658935. doi: 10.3389/fpsyg.2021.658935, PMID: 34122243 PMC8194825

[ref60] Spry A. Pappu R. Cornwell T. B. (2011). Celebrity endorsement, brand credibility and brand equity. Eur. J. Mark. 45, 882–909. doi: 10.1108/03090561111119958

[ref61] Sun M. Jiang L. C. Huang G. (2023). Improving body satisfaction through fitness app use: explicating the role of social comparison, social network size, and gender. Health Commun. 38, 2087–2098. doi: 10.1080/10410236.2022.2054099, PMID: 35350945

[ref62] Thompson J. K. Coovert M. D. Stormer S. M. (1999). Body image, social comparison, and eating disturbance: a covariance structure modeling investigation. Int. J. Eat. Disord. 26, 43–51. doi: 10.1002/(sici)1098-108x(199907)26:1<>3.0.co;2-r, PMID: 10349583

[ref63] Thompson B. Daniel L. G. (1996). Factor analytic evidence for the construct validity of scores: a historical overview and some guidelines. Educ. Psychol. Meas. 56, 197–208. doi: 10.1177/0013164496056002001

[ref64] Tide S. (2022). The home fitness wave and the marketing strategy of fitness brands in the new media era--analysis based on the phenomenon of Frederick Liu. Sports Sci. 43, 19–25. doi: 10.13598/J.Issn1004-4590.2022.05.003

[ref65] Tiggemann M. Mccourt A. (2013). Body appreciation in adult women: relationships with age and body satisfaction. Body Image 10, 624–627. doi: 10.1016/j.bodyim.2013.07.003, PMID: 23954196

[ref66] Tiggemann M. Polivy J. (2010). Upward and downward: social comparison processing of thin idealized media images. Psychol. Women Q. 34, 356–364. doi: 10.1111/j.1471-6402.2010.01581.x

[ref67] Tiggemann M. Williamson S. (2000). The effect of exercise on body satisfaction and self-esteem as a function of gender and age. Sex Roles 43, 119–127. doi: 10.1023/A:1007095830095

[ref68] Wang L. Xu X. Zhang M. Hu C. Zhang X. Li C. . (2023). Prevalence of chronic kidney disease in China: results from the sixth China chronic disease and risk factor surveillance. JAMA Intern. Med. 183, 298–310. doi: 10.1001/jamainternmed.2022.6817, PMID: 36804760 PMC9941971

[ref69] Wang L. Zhou B. Zhao Z. Yang L. Zhang M. Jiang Y. . (2021). Body-mass index and obesity in urban and rural China: findings from consecutive nationally representative surveys during 2004–18. Lancet 398, 53–63. doi: 10.1016/S0140-6736(21)00798-4, PMID: 34217401 PMC7617101

[ref70] Xiang L. Zheng X. Lee M. K. Zhao D. (2016). Exploring consumers’ impulse buying behavior on social commerce platform: the role of parasocial interaction. Int. J. Inf. Manag. 36, 333–347. doi: 10.1016/j.ijinfomgt.2015.11.002

[ref71] Yang Y. Gao J. Qi J. (2024). Moderating effect of consumers’ opinion leader acceptance: exploring the relationship between livestreaming shopping and online shopping safety satisfaction. Electron. Commer. Res., 1–29. doi: 10.1007/s10660-024-09809-6

[ref72] Ye C. Zheng R. Li L. (2022). The effect of visual and interactive features of tourism live streaming on tourism consumers’ willingness to participate. Asia Pac. J. Tour. Res. 27, 506–525. doi: 10.1080/10941665.2022.2091940

[ref73] Ying X. Peng G. Chunqing L. (2021). A study of social presence in live broadcasting: scale development and validity test. Nankai Manag. Rev. 24.

[ref74] Yu J. Song W. (2024). Impact of fitness live streaming on public engagement in physical activities: a cross-sectional study. J. Phys. Educ. Sport. 24, 658–672.

[ref75] Yu L. Tang W. Gao W. (2025). A study on the mechanism of live streamer's behavior characteristics affecting consumers' impulsive buying: the role of perceived value and social identity. Acta Psychol. 255:104950. doi: 10.1016/j.actpsy.2025.104950, PMID: 40157024

[ref76] Yuan B. (2023). Understanding digital information production in virtual communities from the perspective of actor-network theory. J. Hosp. Tour. Manag. 56, 495–502. doi: 10.1016/j.jhtm.2023.08.003

[ref77] Zerfass A. Verčič D. Wiesenberg M. (2016). The dawn of a new golden age for media relations?: how PR professionals interact with the mass media and use new collaboration practices. Public Relat. Rev. 42, 499–508.

[ref78] Zhai X. Wang M. Ghani U. (2023). “The SOR (stimulus-organism-response) paradigm in online learning: an empirical study of students' knowledge hiding perceptions” in Cross reality (XR) and immersive learning environments (Iles) in education. 1st edn. (Routledge), 48–63.

[ref79] Zhang T. Li B. Hua N. (2025). Live-streaming tourism: model development and validations. J. Travel Res. 64, 559–575. doi: 10.1177/00472875231223133

[ref80] Zheng X. Fu S. (2024). Tourism live streaming: uncovering the effects of responsiveness and knowledge spillover on travelling intentions. Tour. Rev. 79, 1126–1146. doi: 10.1108/TR-04-2023-0244

[ref81] Zhou J. Zhou J. Ding Y. Wang H. (2019). The magic of Danmaku: a social interaction perspective of gift sending on live streaming platforms. Electron. Commer. Res. Appl. 34:100815. doi: 10.1016/j.elerap.2018.11.002

